# The effects of social rank and payoff structure on the evolution of group hunting

**DOI:** 10.1371/journal.pone.0269522

**Published:** 2022-06-10

**Authors:** Julie C. Jarvey, Payam Aminpour, Clifford Bohm

**Affiliations:** 1 Department of Integrative Biology, Michigan State University, East Lansing, Michigan, United States of America; 2 Ecology, Evolution, and Behavior Program, Michigan State University, East Lansing, Michigan, United States of America; 3 BEACON Center for the Study of Evolution in Action, Michigan State University, East Lansing, Michigan, United States of America; 4 Department of Community Sustainability, Michigan State University, East Lansing, Michigan, United States of America; Academia Sinica, TAIWAN

## Abstract

Group hunting is common among social carnivores, and mechanisms that promote this behavior are a central topic in evolutionary biology. Increased prey capture success and decreased losses from competitors are often invoked as factors promoting group hunting. However, many animal societies have linear dominance hierarchies where access to critical resources is determined by social rank, and group-hunting rewards are shared unequally. Despite this inequality, animals in such societies cooperate to hunt and defend resources. Game theoretic models predict that rank and relative rewards from group hunting vs. solitary hunting affect which hunting strategies will evolve. These predictions are partially supported by empirical work, but data needed to test these predictions are difficult to obtain in natural systems. We use digital evolution to test how social rank and tolerance by dominants of subordinates feeding while sharing spoils from group hunting influence which hunting strategies evolve in digital organisms. We created a computer-simulated world to reflect social and hunting dynamics of spotted hyenas (*Crocuta crocuta*). We found that group hunting increased as tolerance increased and as the relative payoff from group hunting increased. Also, top-ranking agents were more likely to group hunt than lower-ranking agents under despotic sharing conditions. These results provide insights into mechanisms that may promote cooperation in animal societies structured by dominance hierarchies.

## Introduction

Understanding mechanisms that lead to evolution and maintenance of group hunting in animal societies is a central topic in behavioral and evolutionary ecology and the subject of much theoretical and empirical work (e.g., [1–8]). Group hunting occurs in diverse taxa including birds (e.g., [9–11]), mammals (e.g., [12–14]), fish (e.g., [15,16]), and arachnids (e.g., [17,18]). It is especially common among social carnivores (e.g., [4,19–22]). Group hunting can lead to improved individual energy return through a variety of mechanisms, including increased prey capture success [19,21,23–25], the ability to capture larger prey items when hunting in groups [19,25], and higher success at defending kills against kleptoparasites [6,7,19,23,24,26–29].

Group hunting has many benefits compared to solitary hunting among social carnivores. However, this does not mean that each individual hunter benefits equally. Many carnivore societies are structured by dominance hierarchies and rewards from group hunting are unequally distributed based on dominance rank [19,30–34]. This occurs in spotted hyenas (*Crocuta crocuta*) in which, an individuals’ social rank falls within a strict linear dominance hierarchy that determines its priority of access to critical resources and therefore confers significant fitness advantages to high-ranking individuals [19,30,31,35–37]. Furthermore, like most large gregarious carnivores, spotted hyenas live in fission-fusion societies, such that individuals spend most of their time alone or in small subgroups that change multiple times per day [33]. Therefore, individuals have opportunities to hunt both solitarily and in groups as demanded by ecological and social conditions and thus are not obligate group hunters. Spotted hyenas typically hunt alone for most prey items, and although mean hunting group size is 1.5 hyenas [25], they also commonly hunt in groups to capture larger or more challenging prey [19,25]. Because high-ranking individuals can use aggression to displace subordinates from kills, rewards are unequally distributed, with high-ranking individuals receiving most of the nutritional and energetic rewards from group hunts whereas low-ranking individuals receive little reward [19,31,33]. Thus, rewards for cooperating are unequally distributed based on dominance rank [33,34]. Despite this inequality and these fission-fusion dynamics, individual hyenas, even those with low ranks, still hunt in groups. Research on social cognition has revealed that many animals respond negatively to inequality in reward distribution (reviewed by [38]). Why do low-ranking carnivores participate in group hunts if they receive disproportionately small shares of the hunting rewards or no reward at all?

Because spotted hyenas live in fission-fusion societies in which benefits of collective action are likely to vary with social rank, these societies offer an interesting system in which to investigate the dynamics of group hunting. However, hunting dynamics are difficult to observe in nature and spotted hyenas are too long-lived to study evolution in action. Although empirical data from observational studies provide important information about the factors influencing group hunting, a deeper understanding of the evolution of this phenomenon and individual fitness outcomes is difficult to attain by observing animal behaviors in their natural habitat. Thus, theoretical and computational approaches may yield novel insights into mechanisms promoting group hunting.

Game theory improved our understanding of conditions that facilitate evolution of group hunting. Game theoretical models predict that social rank and relative rewards from cooperative vs solitary hunting affect whether group hunting is an evolutionary stable strategy [2,39]. Although these models have some support from empirical data (reviewed by [2]), the game theoretical framework cannot account for complexities of biological systems such as spatial interactions, stochasticity, mutation rates, and evolutionary dynamics [40]. In contrast, agent-based methods allow for such complexities to be explored and can yield insights into evolutionary dynamics that cannot be achieved by game theory alone [40]. Using computational techniques such as agent-based digital evolution can enhance our understanding of natural systems to help generate and test predictions about collective behavior. Although previous digital evolution experiments have already enhanced our understanding of factors that affect the evolution of cooperative behavior in spotted hyenas (e.g., fluctuating prey availability, communication, and interspecific competitors, [41,42]), variation in social rank has not been considered *in silico* until now.

Here we use digital evolution to examine factors influencing the evolution of group hunting in a society where access to resources is rank-based and individuals have options of hunting alone or in groups. We investigated how social rank and variation, based on differential tolerance among high-ranking hyenas for feeding concurrently with lower-ranking individuals, in the equity with which hunting rewards are shared among group members affect which strategies evolve in agents in a digital system. As also occurs among other animals living in hierarchical societies [43], there is a great deal of variation in how tolerant dominant hyenas are with respect to allowing subordinates access to critical resources [44]. Here we asked specifically: 1) How does tolerance during the sharing of rewards from cooperative hunting affect the evolution of group hunting? 2) Are individuals more likely to group hunt when they are higher- or lower-ranking than other members of their group? We hypothesized that 1) group hunting would decrease or fail to evolve altogether with decreasing tolerance (i.e., increasing rank influence on payoffs) because the per capita payoff from solo hunting would come to outweigh the group-hunting payoff for a greater proportion of agents within the system as tolerance decreases and, 2) higher-ranking individuals would group hunt more often than lower-ranking individuals because they should always receive greater per capita rewards from group hunting.

## Materials and methods

### The game environment

We used a custom-built digital environment created in MABE (Modular Agent Based Evolver) [45] designed to reflect the social structure of spotted hyenas. The digital environment was comprised of a 60 x 60 toroidal grid where each location in the grid was occupied by an agent (3600 agents in total). Agents in the game could not move but could produce offspring after accumulating enough resources to reproduce. Each agent in the initial population was randomly assigned a unique rank, and thereafter, offspring were assigned the rank immediately below that of their parent, reflecting the maternal rank inheritance of spotted hyenas [46]. Rank was used to determine resource distribution after successful group hunts (described below).

#### Hunting games

**Fig 1:**
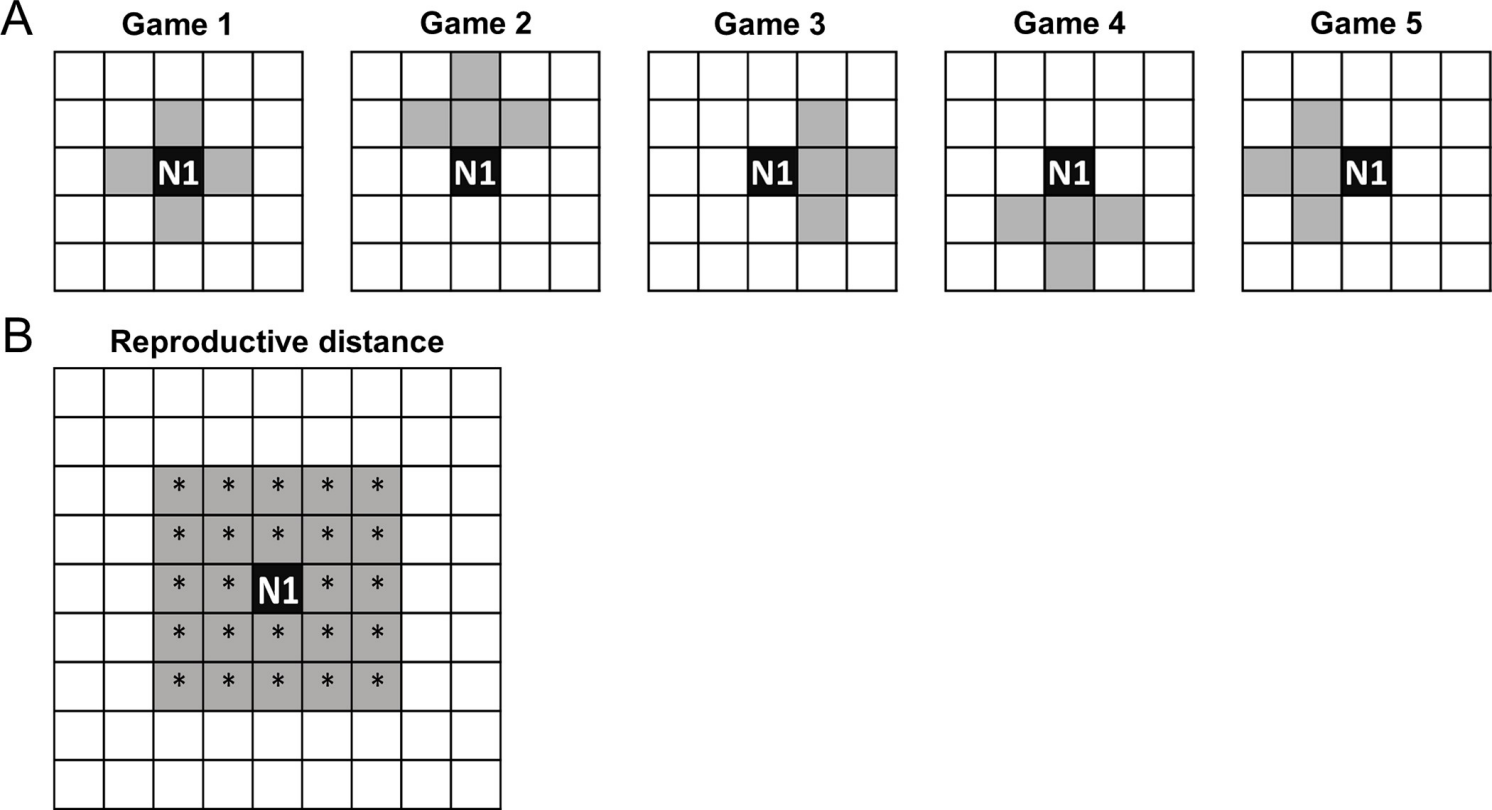
Setup of groups for hunting games and possible offspring agent locations. (A) N1 (black squares) is the location of a single agent. Grey squares represent the neighboring agents that N1 will play the 5 hunting games with during each world update. (B) The locations (grey squares) labeled with * show the possible offspring locations for a parent at location N1 (black square), as we assume offspring remain closer to their parent than do unrelated hyenas. Offspring replace the agent at their location, effectively killing off that agent.

We ran experiments for 1,000,000 updates. Before each world update, each agent played in 5 hunting games in groups with 4 close neighbors. In one game, each agent was the central agent, playing in a game with their immediately adjacent agents, in the other four games, agents were adjacent to the north, south, east, or west of the central agent (Fig 1A). In the hunting games each agent made a single choice, either to group hunt for large prey, or to solo hunt for small prey. If it chose to solo hunt, the agent got the entire small payoff; if it chose to group hunt, they got a larger average per capita payoff, but shared the reward with the other agents that also chose to group hunt. Agents that solo hunted had a 50% chance of receiving the solo-hunt payoff. The success rate for a group hunt was also 50% unless only one agent chose to group hunt; in which case the success rate was 5%. Agents that group hunted had a chance to receive a share of an accumulated group-hunt payoff based on the number of hunters who chose to hunt for large prey. Failed hunts (both solo and group) resulted in no resource gain. These success rates reflect the possibilities 1) that a single individual can choose to hunt a larger prey animal, and 2) that others in the group may not join in the hunt, and 3) the odds of succeeding when spotted hyenas attempt to hunt large prey. Hyenas have a low probability of capturing large prey, such as zebra, when hunting alone, but odds of success increase substantially with a second hunter, although hunting success does not improve much with >2 hunters [25]. We limited our experiments to a system where hunters capture only a single prey item at a time, because we were interested in the binary choice between the consistent payoff of a small prey vs. the payoff of a large prey (where the chance of success depends on whether others choose to cooperate). Additionally, we assumed that the social system of the agents did not evolve due to benefits of group hunting, but rather that group living preceded the evolution of group hunting, which is most likely true for spotted hyenas as it is for lions, in which group hunting was likely a consequence, but not a cause, of the evolution of sociality [2,27].

Agent hunting decisions were determined by values encoded in their 5-site genome. The value at each genome site represented the probability that an agent would group hunt given an agents’ relative rank in its hunting group (e.g., if an agent is the third-ranking agent in the hunting group, the value at the third genome site represents the probability of group hunting in that hunting game).

#### Fitness

An agents’ reproductive success depended on how many resource points it earned from hunting. Before each world update, each agent participated in 5 hunting games. The number of resource points each agent received was equal to the average number of resource points that agent earned from the 5 hunting games. When an agent received enough resource points, it produced an offspring at the next update. The reproduction cost was set at 50 points, the solo-hunt payoff was set at 5, and the group-hunt payoff was variable. Agents accumulated resource points until they achieved enough to reproduce. When they accumulated 50 points, they produced an offspring at the next update, after hunting games were resolved. If agents did not receive enough resource points to reproduce at that update, they carried over their points from previous updates and continued to accumulate points until they could reproduce. When an agent reproduced, it paid the reproduction cost and produced an offspring that was placed randomly into one of the 24 neighboring locations within a 2-grid cell radius of the parent agent (Fig 1B). We choose this design to reflect the fact that female hyenas typically remain in their natal groups throughout their lives [31], and that they associate most closely with their kin [47]. The agent at the new offspring’s location died and any resources it had collected were lost so that the phenotypes reproducing faster would tend to outcompete slower-reproducing phenotypes in the population. The offspring inherited a mutated copy of the parent’s genome and the rank directly below the parents’ rank, following rules of maternal rank inheritance in spotted hyenas [46]. All agents ranking below the new offspring were reassigned to one rank lower to maintain the linear dominance hierarchy. Mutations occurred when new agents were born at a 0.05% probability per genome site. If a mutation occurred, mutated sites were set to a new random probability (i.e., probability of group hunting, in the range [0–1]). After new agents were born, the numeric ranks of all agents in the population were updated to maintain unique rank ordering; that is, the entire population was structured by a linear dominance hierarchy where no two agents had the same rank.

#### Reward structure

**Fig 2:**
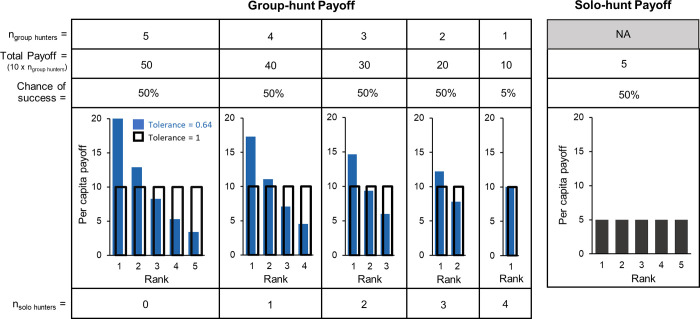
Per capita payoffs under different experimental conditions. Payoff scheme agents received for group hunting and solo hunting under different tolerance conditions (when the group-hunting payoff is 2 times the per capita payoff from solo hunting). Agents hunted in groups with four other agents and each agent decided whether to group hunt (i.e., hunt for large prey and share the reward with other agents that choose to group hunt) or solo hunt (i.e., hunt for small prey and the agent gets a smaller reward that is not shared). If an agent chose to group hunt, the payoff it received depended on its relative rank among other group hunters, the tolerance value, and how many other agents chose to group hunt. Agents received equal shares of the group-hunting payoff when tolerance = 1. As tolerance decreased, the payoff was increasingly skewed by dominance rank, with the highest-ranking agent receiving a greater proportion of the payoff. Agents received the same payoff from solo hunting regardless or rank under all tolerance conditions.

The reward from group hunting was distributed to participating agents based on their relative rank among the group hunters. We manipulated two variables that affected the payoff distribution: tolerance (i.e., how evenly the reward was shared among group hunters based on rank) and the payoff ratio of solo hunts to group hunts. The payoff for a successful solo hunt was 5 points for all experiments. We used two group-hunt payoffs to test the effect of the solo-hunt to group-hunt payoff ratio on which strategies evolve. The group-hunt payoff conditions were set at 2 and 1.2 times the size of the solo-hunt payoff (i.e., per capita average = 10 and 6 points, respectively). The total group-hunt payoff depended on the total number of group hunters so that the average per capita reward remained constant to approximate the relative energy gain individuals would accrue from multiple hunting bouts alone or in a group. We tested five different “tolerance” values: 1.0, 0.96, 0.88, 0.76, 0.64. Tolerance of 1 meant the payoff was shared equally among all agents who group hunted. As tolerance decreased, the payoff became increasingly skewed by social rank. For example, if three agents group hunted and tolerance was 0.64, the second-ranking agent got 64% of the payoff received by the first-ranking agent, and the third-ranking agent got 64% of the payoff received by the second-ranking agent. Note that the rank skew was determined by the relative ranks of group hunters, such that, if only the first-, third-, and fifth-ranking agents choose to group hunt, their ranks for sharing payoffs would be 1, 2, and 3 (Fig 2). We ran simulations for each tolerance factor for the two group-hunt payoff schemes, resulting in 10 total conditions. We ran 200 replicate simulations for each condition for 1,000,000 updates. At each update, we tracked the rate of group hunting based on relative social ranks of agents within each hunting group. We sampled each replicate every 1,000 updates and took the average group-hunt rate of the last 100,000 updates (n = 101 samples). We then took the mean across the 200 replicates to calculate the final mean group-hunt rate for each experimental condition.

All data post-processing, analysis, and plotting were conducting in R [48] version 4.0.5 and RStudio [49] version 2021.9.1.372 using the packages here [50] version 1.0.1, tidyr [51] version 1.2.0, dplyr [52] version 1.0.8, ggplot2 [53] version 3.3.6, and gridExtra [54] version 2.3.

## Results

**Fig 3:**
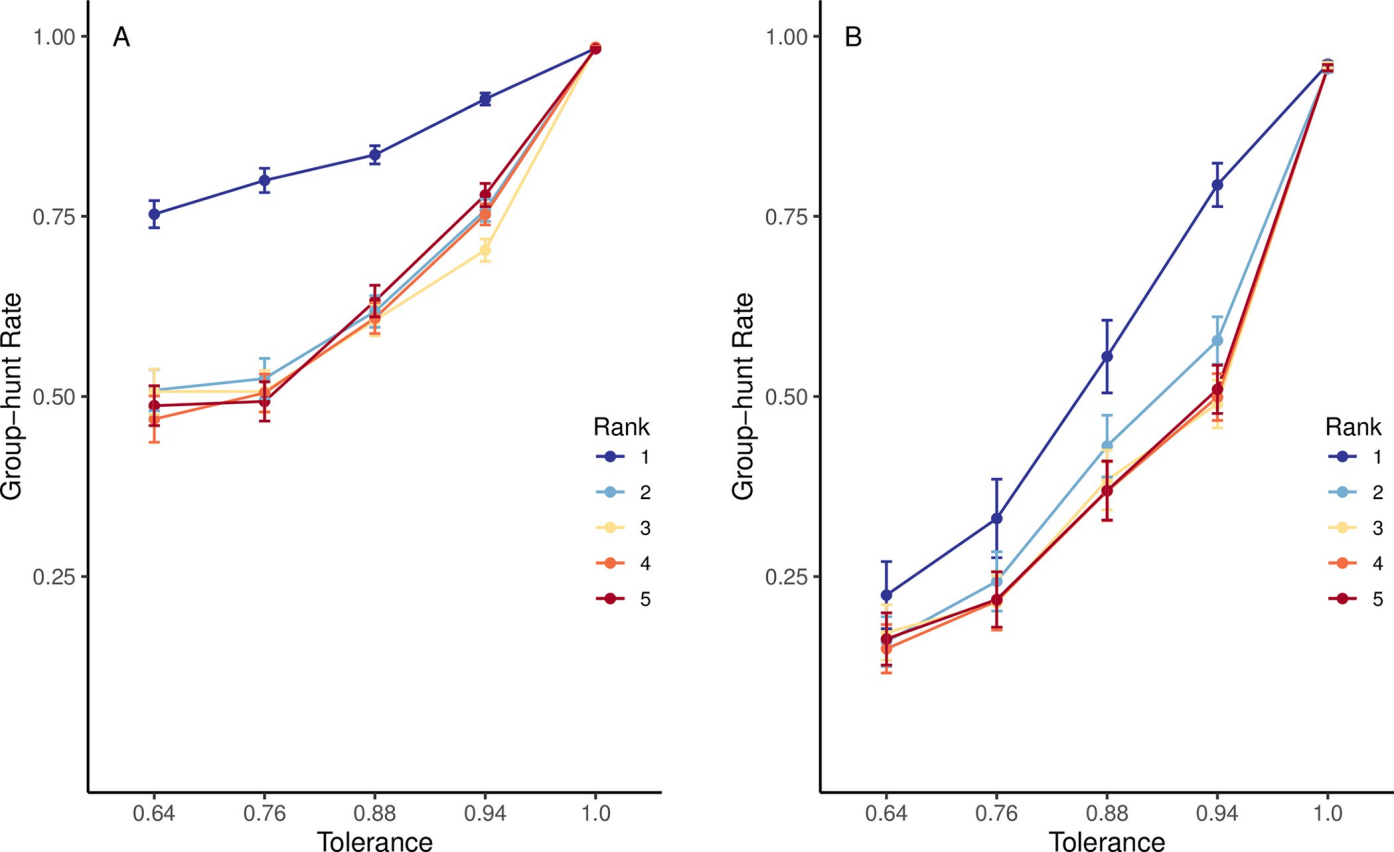
Group-hunting rates under varying tolerance conditions. The average rate of group hunting in the agent population across different tolerance levels when the group-hunting payoff is (A): 2 times the solo-hunt payoff per capita and (B): 1.2 times the solo-hunt payoff per capita. Tolerance determined how equally the reward from group hunting was shared (i.e., 1 = shared equally, 0.64 = each agent gets 64% of the reward as the agent with the rank immediately above it). Each point represents the average group-hunting rate of the agents in their respective relative rank positions within hunting subgroups (e.g., rank 1 = highest ranking agent in the subgroup). Each tolerance condition was run for 1,000,000 updates (sampled every 1,000 updates), plots represent the mean of the last 100,000 updates (n = 101 samples) averaged across 200 replicates per condition. Bars represent ±2 standard errors of the mean group-hunting rates among agents of each relative rank across each tolerance condition.

Group-hunting rates in our population of agents were strongly influenced by the tolerance shown by dominant hyenas (Fig 3). When the reward was divided evenly (i.e., tolerance = 1), agents group hunted nearly 100% of the time under both payoff scenarios. As the reward from group hunting became less evenly distributed (i.e., increasingly skewed by social rank), group-hunting rates decreased within the population.

Hunting decisions were also strongly affected by relative social rank. When tolerance was low, higher-ranking agents generally group hunted at higher rates than did lower-ranking agents. When the group-hunt payoff was twice the magnitude of the solo-hunt payoff, the difference between the group-hunting rates of the highest-ranking agents and all other agents increased with decreasing tolerance, with the greatest difference at the lowest tolerance level of 0.64 (Fig 3A).

The relative size of the group-hunt payoff compared to the solo-hunt payoff had a substantial effect on hunting decisions (Fig 3). When the relative payoff from group hunting was reduced (i.e., from 2 to 1.2 times the solo-hunt payoff), agents group hunted at similar rates only when the reward was divided evenly. However, at all other tolerance levels, group-hunting rates were lower among agents of all relative ranks compared to when the payoff was twice the size of solo hunting. At this smaller group-hunt payoff, the variance in group-hunting rates based on relative social ranks was also reduced (Fig 3B). There were small differences in group-hunting rates among agents of different social ranks at the lowest tolerance levels (0.64 and 0.74). The differences in group-hunting rates between agents with rank 1 and agents with ranks 2 to 5 were larger at moderate tolerance levels (0.88–0.94).

## Discussion

In our agent-based digital evolution system, the evolution of group hunting was strongly influenced by tolerance, social rank, and relative per capita reward from group hunting and solo hunting. These results both support previous game theoretical predictions and emulate findings from empirical studies [2]. Our results also reflect tradeoffs faced by social carnivores.

### Tolerance & social rank

Tolerance during distribution of hunting rewards had a strong effect on group-hunting rates in our experiments. As tolerance decreased, group hunting decreased, especially among lower-ranking agents. These results support previous experimental work. In other species, individuals are sensitive to inequity in rewards for performing the same task [38,55,56]. Mechanisms that increase tolerance in resource sharing and improve benefits for lower-ranking individuals should promote cooperation in societies structured by linear dominance hierarchies. Further work investigating variation in social bonds among individuals could improve our understanding of cooperation in hierarchical societies. High-ranking individuals may share resources more equitably in exchange for other benefits. There is some evidence of such mechanisms in animal societies. For example, low-ranking spotted hyenas gain social and feeding tolerance by associating with high-ranking hyenas [36]. Male chimpanzees share meat with males who are preferred social partners and who provide coalitionary support [57–59]. This increased feeding tolerance may promote participation in cooperative tasks by low-ranking individuals who form stronger bonds with, or provide more social support to, high-ranking individuals. Future work investigating how partner choice, effort, and variation in equity influence participation in cooperative tasks will further our understanding of the conditions under which cooperation evolves [38].

Although group-hunting rates decreased with decreasing tolerance, low-ranking agents still group hunted despite the low payoff these agents received. Kin selection is another mechanism that might promote cooperation and could potentially explain why low-ranking agents sometimes cooperate in highly despotic societies [60]. Individuals that cooperate in groups containing their close relatives receive additional inclusive fitness benefits by helping kin [61]. Furthermore, feeding tolerance can increase when feeding with kin [55], and lower-ranking individuals may gain access to resources through higher-ranking relatives. Our experiments did not test this hypothesis; however, we set the distance offspring were placed in the world from their parent at a maximum of two cells away, meaning that offspring stayed relatively close to their parent, and were likely in one or multiple subgroups with their parent and other relatives. This close physical distance could generate a cluster of closely related agents, all of which inherited similar strategies from their parent agent, and concurrently increase inclusive fitness benefits from cooperating with kin. We expect that setting this reproductive distance farther from the parents would have a negative effect on evolution of cooperation, but more experiments are needed to test this hypothesis. Modifying the distance at which offspring can travel from their parents in digital evolution systems may provide more insights into how kin selection influences group hunting in animal societies.

There may be some conditions under which it is worth cooperating some percentage of time for lower-ranking agents. For example, if the fifth-ranking agent group hunts and the fourth- and third-ranking agents solo hunt, the fifth-ranking agent’s relative rank improves among the group hunters and thus it will receive a better payoff than if all five agents group hunted. Situations such as these may explain why, in our experiments, there was little to no difference between the group-hunt rates of the second- to fifth-ranking agents across tolerance levels under both payoff conditions. Further work is needed to investigate how actions taken by individual agents vary across hunting groups and as their relative rank and the number of group hunters vary in hunting games.

### Reward from solo hunting

The relative per capita reward from group hunting vs. solo hunting also influenced group-hunting rates. As occurs in other species (e.g., [2,24]), we found that when there is greater potential for a higher per capita payoff from group hunting, this strategy should be more common. In our digital system, we kept the group- and solo-hunting success rates consistent throughout the experiments. In natural systems, the group- and solo-hunting success rates can vary spatially and temporally within and among populations due to varying ecological conditions. Chimpanzees in the Gombe forest hunt cooperatively less than chimpanzees in the Taï forest, most likely because solo hunting at Gombe is more successful than solo hunting in Taï [62]. Relative solo- and group-hunting success can also vary within populations due to seasonal changes in prey availability. In the Maasai Mara, Kenya, zebra (*Equus burchelli*) and wildebeest (*Connochaetes taurinus*) abundance peaks for several months during the annual migration [63]. When these large prey are widely available, spotted hyenas have higher hunting success and are more gregarious [25,33], which may affect the relative returns from alternative hunting strategies, particularly for low-ranking individuals. Solo- and group-hunt success rates can also vary with prey type [25], affecting relative payoffs from hunting strategies [2]. For instance, solo-hunting spotted hyenas in the Kalahari Desert have low success hunting adult gemsbok (*Oryx gazella*) but high success hunting calves [64], and thus should profit more by hunting in groups for adults but hunting alone for calves. In contrast, spotted hyenas in Tanzania have greater success when hunting in groups for wildebeest calves but group hunting does not increase hunt success when hunting for adult wildebeest [19]. Future research is needed to understand how varying prey availability and capture success over space and time influence evolutionary dynamics of cooperative hunting.

Our results agree with game theoretical models that the reward from cooperating must be greater than the reward from solo hunting for individuals to hunt cooperatively [2]. In societies where rank determines priority of access to food, this reward must be even larger for low-ranking individuals to cooperate [2,30]. Thus, as solo-hunting success decreases, cooperation should be favored [2]. Here, we tested two group-hunt payoff scenarios; one where the average per capita payoff from group hunting was twice that of the solo-hunt payoff, and one where the average per capita payoff was 1.2 times the solo-hunt payoff. Even when the mean group-hunt per capita payoff was only 1.2 times the solo-hunt payoff, group hunting still occurred, albeit at low rates, under the most despotic conditions for resource sharing. How much inequity will agents tolerate before group hunting disappears from this system?

Social carnivores face a tradeoff between relative costs and benefits gained from solo hunting vs. group hunting. Solitary foragers can be overwhelmed by groups of intra- and inter-specific competitors once a kill has been made, and thus lose control of food resources [21,28], making solo hunting riskier and potentially less profitable than group hunting. However, hunting alone can attract less attention, and successful solo hunters may enjoy more time feeding on their prey before attracting competitors. For example, in the Maasai Mara, on average, 6 or more additional spotted hyenas were present at kills made by pairs within 10 minutes of a successful hunt, whereas hunting alone rarely attracted other hyenas [33]. In our system, we did not include the potential for lost resources due to cheaters or kleptoparasites, but future studies could investigate the relationship between group size and losses due to competitors and how this tradeoff influences individuals’ decisions.

## Conclusions

We conducted a digital evolution experiment to investigate mechanisms promoting group hunting in a social system where reward from cooperative hunting unequally distributed. Although this experiment presents a simplified model of a spotted hyena social system, it demonstrates the utility of agent-based digital evolution in examining complex evolutionary and behavioral dynamics across time scales and ranges of variables that are difficult or impossible to study in natural systems. This method also allows us to manipulate variables and track fitness outcomes to extents that are difficult or impossible to do with living animals. For example, our simulations ran for 1,000,000 updates which approximated roughly 20,000–50,000 generations, representing a time scale that would be impossible to measure in extant long-lived organisms. In this system, we manipulated how rewards were divided among group hunters and the per capita reward agents received from hunting based on hunting strategy, group size, and social rank. In natural systems, observers are limited to natural-occurring behaviors and cannot experimentally manipulate factors this precisely or across these ranges. Thus, digital evolution offers a powerful complement to empirical and theoretical work. In these experiments, we considered the effect of tolerance of the reward from group hunting. However, many other factors can influence the evolution and stability of group hunting such as communication, potential to cheat and punish cheaters, group size, role/effort in hunt, learned behaviors, kin selection, and partner choice [38,65]. Thus, we close with the caveat that other factors merit investigation in future research to elucidate effects of other social and ecological variables. We suggest that doing so will further our understanding of the evolution of group hunting and of cooperative behavior more broadly.

## References

[1] Packer C. Constraints on the evolution of reciprocity: Lessons from cooperative hunting. Ethol Sociobiol. 1988;9: 137–147. doi: 10.1016/0162-3095(88)90018-0

[2] Packer C, Ruttan L. Evolution of cooperative hunting. Am Nat. 1988;132: 159–198.

[3] Mesterton-Gibbons M, Dugatkin LA. Cooperation among unrelated individuals: Evolutionary factors. Q Rev Biol. 1992;67: 267–281.

[4] Stander PE. Cooperative hunting in lions: the role of the individual. Behav Ecol Sociobiol. 1992;29: 445–454. doi: 10.1007/BF00170175

[5] Watts DP, Mitani JC. Hunting behavior of Chimpanzees at Ngogo, Kibale National Park, Uganda. Int J Primatol. 2002;23: 1–28. doi: 10.1023/A:1013270606320

[6] Vucetich JA, Peterson RO, Waite TA. Raven scavenging favours group foraging in wolves. Anim Behav. 2004;67: 1117–1126. doi: 10.1016/j.anbehav.2003.06.018

[7] Carbone C, Frame L, Frame G, Malcolm J, Fanshawe J, Fitzgibbon C, et al. Feeding success of African wild dogs (*Lycaon pictus*) in the Serengeti: the effects of group size and kleptoparasitism. J Zool. 2005;266: 153–161. doi: 10.1017/S0952836905006710

[8] Tennie C, Gilby IC, Mundry R. The meat-scrap hypothesis: Small quantities of meat may promote cooperative hunting in wild chimpanzees Pan troglodytes. Behav Ecol Sociobiol. 2009;63: 421–431. doi: 10.1007/s00265-008-0676-3

[9] Hector DP. Cooperative hunting and its relationship to foraging success and prey size in an avian predator. Ethology. 1986;73: 247–257. doi: 10.1111/j.1439-0310.1986.tb00915.x

[10] Bednarz JC. Cooperative hunting in Harris’ Hawks (*Parabuteo unicinctus*). Science (1979). 1988;239: 1525–1527. doi: 10.1126/science.239.4847.1525 17772751

[11] Yosef R, Yosef N. Cooperative hunting in Brown-necked Raven (*Corvus rufficollis*) on Egyptian Mastigure (*Uromastyx aegyptius*). J Ethol. 2010;28: 385–388. doi: 10.1007/s10164-009-0191-7

[12] Busse C. Do chimpanzees hunt cooperatively? Am Nat. 1978;112: 767–770.

[13] Benoit-Bird KJ, Au WWL. Cooperative prey herding by the pelagic dolphin, Stenella longirostris. J Acoust Soc Am. 2009;125: 125–137. doi: 10.1121/1.2967480 19173400

[14] Pitman RL, Durban JW. Cooperative hunting behavior, prey selectivity and prey handling by pack ice killer whales (Orcinus orca), type B, in Antarctic Peninsula waters. Mar Mamm Sci. 2012;28: 16–36. doi: 10.1111/j.1748-7692.2010.00453.x

[15] Schmitt RJ, Strand SW. Cooperative foraging by yellowtail, *Seriola lalandei* (Carangidae), on two species of fish prey. Copeia. 1982;1982: 714–717. doi: 10.2307/1444679

[16] Strübin C, Steinegger M, Bshary R. On group living and collaborative hunting in the yellow saddle goatfish (*Parupeneus cyclostomus*). Ethology. 2011;117: 961–969. doi: 10.1111/j.1439-0310.2011.01966.x

[17] Rypstra AL. Aggregations of *Nephila clavipes* (L.) (Araneae, Araneidae) in relation to prey availability. Am Arachnol Soc. 1985;13: 71–78.

[18] Tizo-Pedroso E, Del-Claro K. Cooperation in the neotropical pseudoscorpion, *Paratemnoides nidificator* (Balzan, 1888): feeding and dispersal behavior. Insectes Soc. 2007;54: 124–131. doi: 10.1007/s00040-007-0931-z

[19] Kruuk H. The spotted hyena: a study of predation and social behavior. Chicago: University of Chicago Press; 1972.

[20] Bowen WD. Variation in coyote social organization: the influence of prey size. Can J Zool. 1981;59: 639–652. doi: 10.1139/z81-094

[21] Fanshawe JH, Fitzgibbon CD. Factors influencing the hunting success of an African wild dog pack. Anim Behav. 1993. pp. 479–490. doi: 10.1006/anbe.1993.1059

[22] Bailey I, Myatt JP, Wilson AM. Group hunting within the Carnivora: physiological, cognitive and environmental influences on strategy and cooperation. Behav Ecol Sociobiol. 2013;67: 1–17. doi: 10.1007/s00265-012-1423-3

[23] Stander PE, Albon SD. Hunting success of lions in a semi-arid environment. Symp Zool Soc London. 1993; 127–143.

[24] Creel S, Creel NM. Communal hunting and pack size in African wild dogs, Lycaon pictus. Anim Behav. 1995;51: 499.

[25] Holekamp KE, Smale L, Berg R, Cooper SM. Hunting rates and hunting success in the spotted hyena. J Zool. 1997;242: 1–15.

[26] Caraco T, Wolf L. Ecological determinants of group sizes of foraging lions. Am Nat. 1975;109: 343–352.

[27] Packer C. The ecology of sociality in felids. In: In: Rubenstein DI, Wrangham RW, editors. Ecological aspects of social evolution. Princeton: Princeton University Press; 1986. pp. 429–451.

[28] Cooper SM. Optimal hunting group size: the need for lions to defend their kills against loss to spotted hyaenas. Afr J Ecol. 1991;29: 130–136. doi: 10.1111/j.1365-2028.1991.tb00993.x

[29] Périquet S, Valeix M, Claypole J, Drouet-Hoguet N, Salnicki J, Mudimba S, et al. Spotted hyaenas switch their foraging strategy as a response to changes in intraguild interactions with lions. J Zool. 2015;297: 245–254. doi: 10.1111/jzo.12275

[30] Tilson RL, Hamilton WJ. Social dominance and feeding patterns of spotted hyaenas. Anim Behav. 1984;32: 715–724. doi: 10.1016/S0003-3472(84)80147-5

[31] Frank LG. Social organization of the spotted hyaena *Crocuta crocuta*. II. Dominance and reproduction. Anim Behav. 1986;34: 1510–1527.

[32] Atwood TC, Gese EM. Coyotes and recolonizing wolves: social rank mediates risk-conditional behaviour at ungulate carcasses. Anim Behav. 2008;75: 753–762. doi: 10.1016/j.anbehav.2007.08.024

[33] Smith JE, Kolowski JM, Graham KE, Dawes SE, Holekamp KE. Social and ecological determinants of fission-fusion dynamics in the spotted hyaena. Anim Behav. 2008;76: 619–636. doi: 10.1016/j.anbehav.2008.05.001

[34] Gese EM, Ruff RL, Crabtree RL. Foraging ecology of coyotes (*Canis latrans*): The influence of extrinsic factors and a dominance hierarchy. Can J Zool. 1996;74: 679–783.

[35] Holekamp KE, Smale L, Szykman M. Rank and reproduction in the female spotted hyaena. J Reprod Fertil. 1996;108: 229–237. doi: 10.1530/jrf.0.1080229 9038781

[36] Smith JE, Memenis SK, Holekamp KE. Rank-related partner choice in the fission-fusion society of the spotted hyena (*Crocuta crocuta*). Behav Ecol Sociobiol. 2007;61: 753–765. doi: 10.1007/s00265-006-0305-y

[37] Holekamp KE, Strauss ED. Reproduction within a hierarchical society from a female’s perspective. Integr Comp Biol. 2020;60: 753–764. doi: 10.1093/icb/icaa068 32667986

[38] Brosnan SF, de Waal FBM. Evolution of responses to (un)fairness. Science (1979). 2014;346. doi: 10.1126/science.1251776 25324394 PMC4451566

[39] Axelrod R, Hamilton WD. The evolution of cooperation. Science (1979). 1981;211: 1390–1396. doi: 10.1126/science.7466396 7466396

[40] Adami C, Schossau J, Hintze A. Evolutionary game theory using agent-based methods. Phys Life Rev. 2016;19: 1–26. doi: 10.1016/j.plrev.2016.08.015 27617905

[41] Rajagopalan P, Rawal A, Miikkulainen R, Wiseman MA, Holekamp KE. The role of reward structure, coordination mechanism and net return in the evolution of cooperation. 2011 IEEE Conf Comput Intell Games, CIG 2011. 2011; 258–265. doi: 10.1109/CIG.2011.6032015

[42] Rajagopalan P, Holekamp KE, Miikkulainen R. Factors that Affect the Evolution of Complex Cooperative Behavior. In ALIFE 2019: The 2019 Conference of Artificial Life 2019 Jul 1 (pp. 333–340). MIT Press doi: 10.1162/isal_a_00184.xml

[43] Sapolsky RM, Share LJ. A pacific culture among wild baboons: Its emergence and transmission. PLoS Biol. 2004;2: e106. doi: 10.1371/journal.pbio.0020106 15094808 PMC387274

[44] Smith JE, Estrada JR, Richards HR, Dawes SE, Mitsos K, Holekamp KE. Collective movements, leadership and consensus costs at reunions in spotted hyaenas. Anim Behav. 2015;105: 187–200. doi: 10.1016/j.anbehav.2015.04.023

[45] Bohm C, Nitash CG, Hintze A. MABE (Modular Agent Based Evolver): A Framework for Digital Evolution Research. Proc Eur Conf Artif Life. 2017; 4–8.

[46] Engh AL, Esch K, Smale L, Holekamp KE. Mechanisms of maternal rank “inheritance” in the spotted hyaena, *Crocuta crocuta*. Anim Behav. 2000;60: 323–332. doi: 10.1006/anbe.2000.1502 11007641

[47] Holekamp KE, Cooper SM, Katona CI, Berry NA, Frank LG, Smale L. Patterns of association among female spotted hyenas (*Crocuta crocuta*). J Mammal. 1997;78: 55–64.

[48] R Core Team. R: A language and environment for statistical computing. R Foundation for Statistical Computing, Vienna, Austria.; 2021. https://www.R-project.org/.

[49] RStudio Team. RStudio: Integrated Development Environment for R. RStudio, PBC, Boston, MA; 2021. http://www.rstudio.com/.

[50] Müller K. here: A Simpler Way to Find Your Files. R package version 1.0.1. 2020. https://CRAN.R-project.org/package=here.

[51] Wickham H, Girlich M. tidyr: Tidy Messy Data. R package version 1.2.0. 2022. https://CRAN.R-project.org/package=tidyr.

[52] Wickham H, François R, Henry L, Müller K. dplyr: A Grammar of Data Manipulation. R package version 1.0.8. 2022. https://CRAN.R-project.org/package=dplyr.

[53] Wickham H. ggplot2: Elegant Graphics for Data Analysis. Springer-Verlag New York; 2016.

[54] Auguie B. gridExtra: Miscellaneous Functions for “Grid” Graphics. R package version 2.3. 2017. https://CRAN.R-project.org/package=gridExtra.

[55] de Waal FBM, Davis JM. Capuchin cognitive ecology: cooperation based on projected returns. Neuropsychologia. 2003;41: 221–228. doi: 10.1016/s0028-3932(02)00152-5 12459220

[56] Wascher CAF, Bugnyar T. Behavioral responses to inequity in reward distribution and working effort in crows and ravens. PLoS ONE. 2013;8: e56885. doi: 10.1371/journal.pone.0056885 23437262 PMC3577644

[57] Mitani JC, Watts DP. Why do chimpanzees hunt and share meat? Anim Behav. 2001;61: 915–924. doi: 10.1006/anbe.2000.1681

[58] Nishida T, Hasegawa T, Hayaki H, Takahata Y, Uehara S. Meat-sharing as a coalition strategy by an alpha male chimpanzee? Top Primatol. 1992;1: 159–174.

[59] Samuni L, Preis A, Mielke A, Deschner T, Wittig RM, Crockford C. Social bonds facilitate cooperative resource sharing in wild chimpanzees. Proc R Soc B Biol Sci. 2018;285: 20181643. doi: 10.1098/rspb.2018.1643 30305438 PMC6191705

[60] Shimoji H, Dobata S. The build-up of dominance hierarchies in eusocial insects. Philos Trans R Soc Lond B Biol Sci. 2022;377: 20200437. doi: 10.1098/rstb.2020.0437 35000446 PMC8743887

[61] Hamilton WD. The genetical evolution of social behavior II. J Theor Biol. 1964. pp. 17–52. doi: 10.1016/0022-5193(64)90039-6 5875340

[62] Boesch C. Cooperative hunting in wild chimpanzees. Anim Behav. 1994. pp. 653–667. doi: 10.1006/anbe.1994.1285

[63] Cooper SM, Holekamp KE, Smale L. A seasonal feast: long-term analysis of feeding behaviour in the spotted hyaena (*Crocuta crocuta*). Afr J Ecol. 1999;37: 149–160. doi: 10.1046/j.1365-2028.1999.00161.x

[64] Mills MGL. Related spotted hyenas forage together but do not cooperation in rearing young. Nature. 1985;316: 61–62. doi: 10.1038/316507a0

[65] Campbell MW, Watzek J, Suchak M, Berman SM, de Waal FBM. Chimpanzees (*Pan troglodytes*) tolerate some degree of inequity while cooperating but refuse to donate effort for nothing. Am J Primatol. 2020;82: 1–14. doi: 10.1002/ajp.23084 31894611 PMC6989098

